# Feasibility and acceptability of a peer-led HIV self-testing model among female sex workers in Malawi: a qualitative study

**DOI:** 10.1136/bmjopen-2021-049248

**Published:** 2021-12-30

**Authors:** Moses K Kumwenda, Webster Mavhu, Wezzie S Lora, Richard Chilongosi, Simon Sikwese, Miriam Taegtmeyer, Karin Hatzold, Cheryl C Johnson, Elizabeth L Corbett, Nicola Desmond

**Affiliations:** 1 Malawi-Liverpool-Wellcome Trust Clinical Research Programme, Blantyre, Malawi; 2 Centre for Sexual Health and HIV/AIDS Research (CeSHHAR) Zimbabwe, Harare, Zimbabwe; 3 Department of International Public Health, Liverpool School of Tropical Medicine, Liverpool, UK; 4 Population Services International Malawi, Blantyre, Malawi; 5 Pakachere Institute for Health and Development Communication, Blantyre, Malawi; 6 Tropical Infectious Diseases Unit, Royal Liverpool University Hospital, Liverpool, UK; 7 Population Services International, Washington, District of Columbia, USA; 8 Department of HIV, World Health Organization, Geneve, Switzerland; 9 Clinical Research Department, London School of Hygiene & Tropical Medicine, London, UK

**Keywords:** HIV & AIDS, public health, qualitative research

## Abstract

**Objectives:**

HIV testing is the gateway to HIV prevention and care services. Female sex workers (FSW) may benefit from HIV self-testing (HIVST), which offers greater control and confidentiality than other approaches. However, FSW also have unique vulnerabilities, making it critical to understand their perspective of HIVST to best contextualise HIVST to their needs. This study explored feasibility and acceptability of providing oral fluid-based peer-led HIVST to FSW to inform tailored HIVST delivery approaches.

**Design:**

Qualitative study.

**Setting:**

Malawi.

**Participants:**

Thirty-nine FSW who had obtained a HIVST kit and eight peer distributors.

**Results:**

Peer distributors’ accounts suggested that peer-led HIVST is feasible. Overall, FSW spoke positively about peer-led HIVST and younger FSW preferred it to facility-based HIV testing. FSW highlighted both greater control of their testing experience and that HIVST could allow them to avoid discriminatory attitudes frequently experienced in public facilities. Some also felt that HIVST kits could enable them to establish the HIV status of their sexual partners, better informing their decisions about condomless sex. Despite overall acceptance of HIVST, a few expressed doubts in the procedure. Some FSW already aware of their HIV-positive status reported using HIVST. A few accounts suggested peer pressure to self-test predominantly from peer distributors.

**Conclusions:**

This study enabled us to explore feasibility and acceptability of peer-led HIVST among FSW, as well as potential shortcomings of the HIV testing modality. Peer distributors are a welcome additional model. However, they should avoid distribution in actual venues. Programmes should ensure a range of testing options are available and expand peer’s representation. Study findings will be used to tailor the HIVST distribution model to ensure its enhanced uptake among key populations in general and FSW, specifically.

**Strengths and limitations of this study**

- This first HIV self-testing study among female sex workers (FSW) in Malawi was integrated within an established FSW service.
- There was concordance between our study findings and those from other studies, including around FSW’s low rates of linkage to treatment, which suggests that the context in Malawi is not unique.
- We used convenience and snowball sampling to select respondents, which, while more feasible in populations with potentially stigmatised behaviours, may not be universally representative.
- Given the fluidity of sex work typologies of street or venue-based FSW in this population, it was not possible to attribute study findings to specific typologies.
- We had intended to reinterview all in-depth interview respondents after 3 months but only managed to reach 28% due to FSW’s recognised mobility.

## Introduction

Despite the global decline in HIV infections in general populations, both HIV incidence and prevalence among female sex workers (FSW) remain unacceptably high.^1–3^ Globally, the risk of HIV acquisition in FSW aged 15–49 years is 26 times higher than that of other women within the same age bracket.^3^ HIV testing is an essential entry point into HIV prevention, treatment and care.^4–7^ Initiatives to increase access to, and uptake of, HIV testing include provider-initiated HIV testing and counselling,^8^ community-based approaches^9^ and partner notification.^10^ Despite these initiatives, a substantial HIV testing gap remains, and in many settings, HIV testing continues to miss some population groups including FSW.^1^ As with other key population groups, FSW experience pervasive and multifactorial structural barriers to conventional HIV testing services including community-level and institutional-level stigma and discrimination.^3 6 11^ It is, therefore, critical to explore innovative ways to ensure equitable access to HIV testing services for FSW.

In sub-Saharan Africa (SSA), HIV self-testing (HIVST), a relatively recent testing approach, is showing potential to overcome FSW’s barriers to conventional HIV testing services.^2 11–13^ The public health assumption is that for key populations in general, and FSW in particular, HIVST’s potential benefits may include the possibility of frequent testing and reduced sexual risk behaviour.^14^ Malawi FSW have previously been categorised as either ‘bargirls’ (venue-based) or ‘freelance’ (street/home based), with the latter largely in charge of their own transactions and working independently of venues.^15^ However, recent research established that these categories are not necessarily fixed as FSW often belong to more than one category and/or transition between categories.^16^ Further, as a highly mobile group, FSW may be venue-based in one locale but may change to street-based in other locales.^16^ Understanding FSW’s contexts, experiences and perspectives of HIVST is critical for contextualising HIVST delivery modalities to their needs.

We report on a qualitative evaluation of a pilot project exploring feasibility and acceptability of providing oral fluid-based peer-led HIVST to FSW in an urban setting in Malawi to inform adaptation of HIVST delivery approaches to this group’s needs.

## Methods

### Study setting

**Figure 1:**
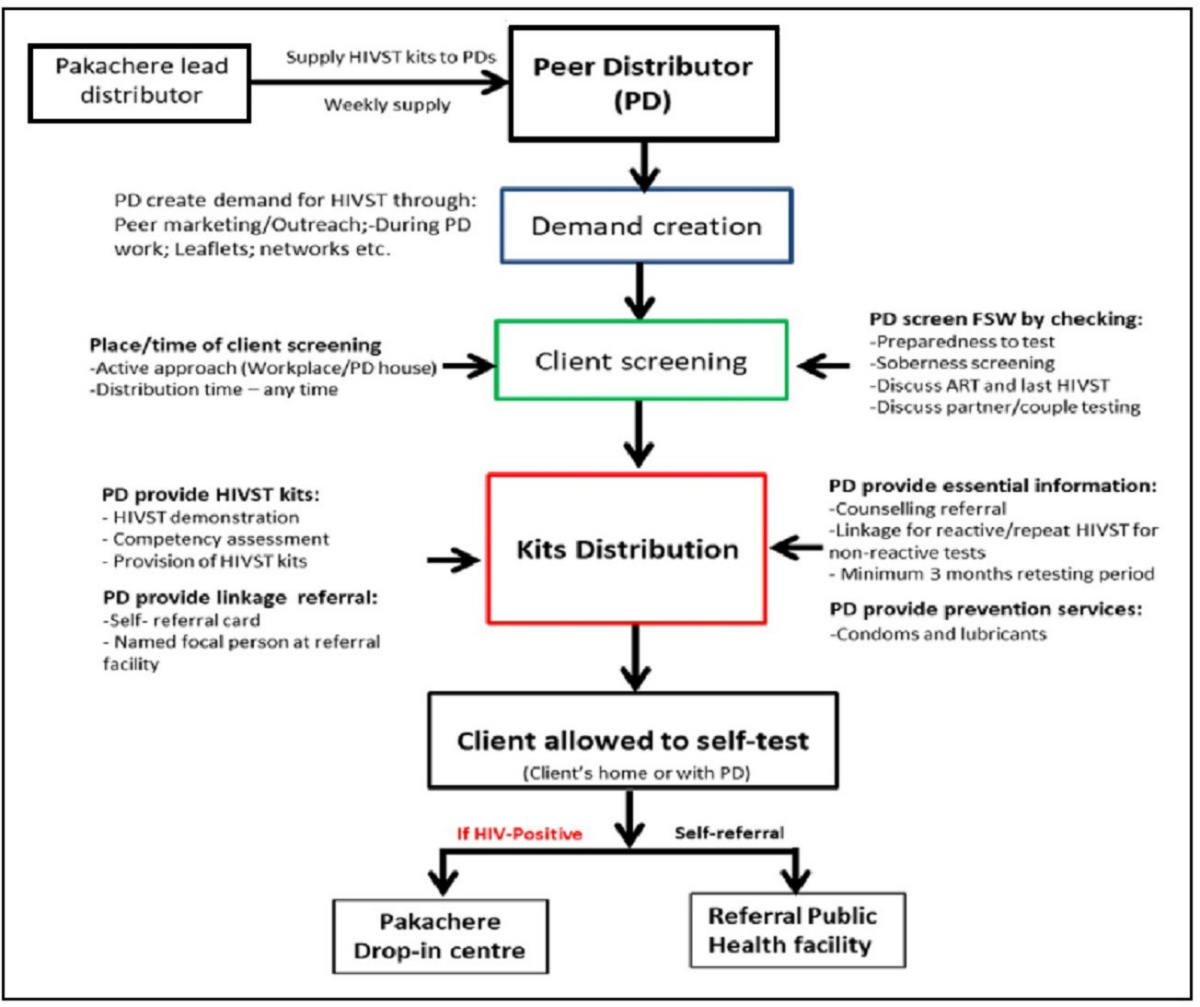
HIVST delivery model. ART, antiretroviral therapy; HIVST, HIV self-testing.

This study was embedded within the Self-Test Africa project (https://www.psi.org/project/star/) implemented by Population Services International and Malawi Liverpool Wellcome Trust Clinical Research Programme (https://www.mlw.mw/) working with Pakachere (https://www.pakachere.org). Over 6 months, the project offered HIVST to FSW through peer distributors in three southern districts of Malawi: Blantyre, Chikhwawa and Mulanje. In these districts, Pakachere was already providing FSW sexual and reproductive health services including provider-delivered, blood-based HIV testing. Informed by formative work,^2^ which suggested that FSW’s preference was for distribution of HIVST kits through peers rather than through the broader community, Pakachere invited FSW to identify peers they trusted to serve as peer distributors (PDs). PDs were then trained to provide pretest and post-test counselling, respond to frequently asked questions, offer oral fluid-based HIVST, and link those with a reactive result to post-test services (ie, confirmatory testing, treatment initiation) (figure 1).

### Study design and respondents

A pilot project offered peer-based HIVST to FSW from February to July 2017. Between February and November 2017, we conducted a qualitative process evaluation that was complementary to a quantitative study where 131 participants were surveyed at two time-points to assess HIVST use and experiences.^17^ Using convenience and snowball sampling, 18 FSW who had obtained a HIVST kit were invited for in-depth interviews (IDIs) within a week of self-testing and 5/18 (28%) were interviewed again after 3 months. Initially, four FSW (n=2 street-based; n=2 predominantly venue based) were identified. They were tasked to refer additional participants to the study and the chain continued until we had 18 respondents (n=12 predominantly venue based) (table 1).

**Table 1:** Respondents’ demographic characteristics (N=18)

Characteristic	n (%)
Age	
15–19 years	5 (27.8)
20–24 years	9 (50.0)
25–29 years	4 (22.2)
No of years in sex work	
1–3 years	15 (83.3)
4–6 years	2 (11.1)
≥7 years	1 (5.6)
Predominantly venue based?	
Yes	12 (66.7)
No	6 (33.3)
HIV-positive status known already?	
Yes	2 (11.1)
No	7 (38.9)
HIV-negative	9 (50.0)

IDIs were chosen for this study to allow individual participants’ experiences to be explored in detail. To triangulate IDIs, three focus group discussion (FGD) were held with an additional 21 FSW 3 months after project implementation. These were purposively selected from PDs’ registers, taking into consideration place of sex work. A separate FGD was also held with eight peer distributors. For all study participants, it was their first time to learn about HIVST.

### Patient and public involvement

FSWs were study participants in the formative work^2^ that informed the pilot project’s peer distribution model. Formative work activities included rapid ethnographic assessments and a stakeholder participatory workshop where FSW helped refine the peer distribution model which this qualitative study explored. Additionally, peer distributors offered peer-based HIVST during the pilot project and were qualitative study respondents. Preliminary study findings were presented to selected respondents for validation purposes. Final study results were orally presented to respondents and FSW groups during allied project and outreach activities.

### Theoretical paradigm and positionality

Both data collection and analysis were largely guided by interpretivism, a research paradigm that acknowledges the importance of understanding people in terms of their own definition and within their own contexts and in their natural settings.^18^ As educated, privileged, multiracial researchers with limited or no experience of sex work, we discussed our potential biases whether conscious or unconscious and reflected on how they might impact interpretation. For example, due to prior experiences, our team could easily assume that HIVST was acceptable and only focus on its delivery yet as illustrated later, there were nuances around acceptability. We, therefore, strived to understand perceptions and experiences of peer-led HIVST among FSW within the lens of their own interpretations, shared meanings and lived experiences.

### Data collection and analysis

The IDIs and FGDs took place in a private space and were facilitated by two male, experienced and trained researchers. Both interviewers were qualified social scientists with additional training in postgraduate qualitative research methods, good clinical practice and the study protocol/procedures. They had only met PDs during some trainings but not the other participants.

All discussions were informed by a topic guide (see online supplemental file) and held in Chichewa, the respondents’ language. Discussions explored issues relating to FSW’s broader life narratives; the type of sex work they undertook; how HIVST aligned with different types of partners; the peer-led model; oral fluid-based HIVST and linkage to treatment. With the participants’ permission, all discussions were audio-recorded and hand-written notes taken. IDIs and FGDs lasted approximately 1 and 2 hours, respectively.

10.1136/bmjopen-2021-049248.supp1 Supplementary data

Audiorecorded data were transcribed and translated verbatim into English by an experienced team. The team also wrote an interview summary following each IDI. The interview summary was both a descriptive and analytic synopsis of the IDI and included reflections of both its conduct and emerging issues (eg, whether use of humour helped ‘normalise’ a difficult conversation). Iterative data collection and analysis was conducted, in which the team discussed each interview in weekly analytical meetings. This informed the refinement of the topic guides. The summaries and weekly analytical meetings informed development of a provisional coding framework.

Half of the discussions (n=9 IDIs, n=2 FGDs) were then independently coded by two male researchers (WM and MK) using the coding framework. WM was only engaged at this stage to minimise the risk of biased interpretations. Discrepancies between WM and MK were resolved through discussion with two female researchers (ND and WSL) until consensus was reached. Of note, WSL (young, Malawian) had previously conducted ethnographic assessments in sex work venues; her experiences provided context to some findings and enriched the analysis. Any additional codes identified from this first set of transcripts were added to the coding framework. Transcripts were entered into NVivo V.12 (QSR International, Melbourne, Australia). Transcripts were then fully coded using the modified coding framework; care was taken to identify any additional emerging codes. Codes were grouped and emerging themes were identified using thematic analysis.^19^

## Results

### IDI respondents’ characteristics

We conducted IDIs with 18 FSW (table 1). Participants’ mean age was 22 years and the average number of years in sex work was three. At time of interviews, 12/18 (67%) were predominantly venue based. Of 18, 9 (50%) reported that their HIVST result was negative. Among those who reported an HIV-positive result, 7/9 (78%) said this was the first time they had become aware; the other two had already tested positive previously.

Discussions with peer distributors (PDs) suggested that peer-led HIVST is a feasible and acceptable testing approach among FSW. FSW highlighted how HIVST had facilitated their access to HIV testing and described how the decision to test was influenced by peers. Despite overall acceptance of HIVST, a few FSW expressed doubts in the procedure. Further, FSW described their different partners and the potential role of being able to access HIVST kits during selected partnerships. At time of interviews, only a few HIV-positive FSW had accessed treatment. We present these themes in more depth below.

### HIVST and HIV testing behaviour

Overall, FSW described suboptimal access to targeted HIV testing prior to this intervention, with most saying they had previously only tested as part of mandatory pregnancy-related testing. Other testing experiences were mostly related to frequent illness. *‘I was getting ill frequently and this led me to go for tests’* (IDI, 02). For those who had previously procrastinated in engaging with provider-delivered blood-based testing (which involves a finger prick), oral fluid-based HIVST enabled them to eventually overcome their general fears around HIV testing. *‘Not so long ago, I was ill. I went to Queen’s hospital and they advised me to get tested for HIV but I did not do so. But when a colleague told me that there were some ‘things’ that were used to test HIV using saliva, I decided to test myself’* (IDI, 04). On the whole, respondents liked that oral fluid-based HIVST was not invasive and did not involve taking blood (considered painful).

### Influence of peer relations on testing

Accounts highlighted the influence of social relations on testing when some respondents noted that even with HIVST, they would not have managed to overcome their fears without peer support. In many instances, the decision to test was influenced by peers. *‘…What happened is that this friend of mine started to lose weight abnormally and she looked weak. So, I approached her and told her to go for testing. She went and tested positive [HIV]’* (PD, FGD).

In addition, descriptions of the HIVST process suggested that, without peer support or the support of a PD, some FSW may not have accurately tested themselves. This was in part due to literacy challenges. *‘*…*I cannot read very well and so she was helping me including with interpreting the result’* (IDI. 13). Others felt that they could not test on their own as the process of performing a self-test was perceived as complex. *‘I was doing it and she [PD] was instructing me since I had never done it before’* (IDI. 14). Clearly, self-testing with peer-support had the potential to result in inadvertent disclosure of a positive result, although this was rarely considered problematic.

Some accounts however, insinuated that a peer’s (ie, a PD or another FSW) mere presence exerted some pressure to self-test. An FSW who had recently tested for HIV described how she had accepted the HIVST kit and went on to self-test largely to ‘please’ the PD. *‘Aah, I knew I was ‘fine’ [HIV negative] but I didn’t want to disappoint the one that gave me the testing tool. So, I just wanted to test and ‘give’ her the result’* (IDI. 03). Evidently, the peer’s presence sometimes resulted in tacit coercion into self-testing or receiving a self-test kit.

Discussions suggested that peer pressure to self-test may have been mostly felt by venue-based FSW, with respondents professing knowledge of who had or had not self-tested. *‘All of us have now tested. There was only one who had not tested but she too has now tested’* (IDI. 05). Venue owners may also have played a part. Describing her previous experience, a FSW remarked, *‘…Since we were ‘sleeping’ [having transactional sex] with married men, the condom could possibly burst…So the ‘boss’ took all of us girls to the hospital to get us tested. Once one was found positive [HIV], she was not allowed to continue working’* (IDI. 02). However, overall, the influence of social relationships on decisions around testing was generally not perceived as negative but rather as a positive or benign encouragement.

### Access: HIVST versus facility-based HIV testing

FSW spoke positively about peer-led HIVST. They preferred this approach to facility-based testing and described it as confidential, convenient, and flexible. Importantly, FSW felt HIVST enabled them to avert the discriminatory treatment they experienced when accessing HIV testing services. ‘*Sex workers are perceived as ‘dogs’. When we go to a public facility they say, ‘Can you step aside? We would like to help the other people’’* (IDI, 14). Accounts suggested that clinic staff were even more judgemental when dealing with younger FSW. *‘Of course, I do sex work, instead of talking about the reason I was there [at clinic], they started focusing on my age. ‘A child like you, eeh this and that…I didn’t go there again’’* (IDI, 14–17 year-old).

FSW also felt that facility-based HIV testing had the potential to either suggest or reveal that they were HIV positive and therefore, had the potential to scare away clients and compromise their livelihood. *‘…Some of the men who intended to ‘sleep’ with you will become afraid that this woman is positive, yaa’* (IDI, 04). Evident in most accounts was community-wide stigmatising attitudes towards FSW.

FSW felt that compared with HIVST, facility-based HIV testing was more costly. ‘*This [HIV self-testing] found us right here while with the other one, you have to travel and spend money’* (IDI, 05). In this study, respondents also noted HIVST’s comparative advantages in relation to opportunity cost. *‘…I see the kit as a good thing because it doesn’t take much time unlike going to the hospital and be moving with the queues…’* (IDI, 10). As facility-based testing is offered during the day (the time FSW want to sleep), FSW appreciated that HIVST kits can both be distributed at night and at venues they frequent (eg, bars).

Despite overall high preference of HIVST over facility-based testing, a few respondents expressed doubts over the reliability of oral fluid-based HIVST, partly due to failure to appreciate the testing mechanism. *‘…We had challenges when one was doing testing and she was waiting for the result and saw the red stuff she was like ‘Aah, is this true? I swabbed my mouth which only has saliva but now I see ‘blood’ [red liquid in test stick] in here’* (PD, FGD). A few FSW described seeking facility-based confirmatory testing regardless of the HIVST result due to disbelief in the result. An FSW who reportedly tested HIV negative explained why she sought a second test. *‘*…*To confirm that it is indeed true, to truly believe’* (IDI, 04). Although a confirmatory test is recommended anyway for an initial HIV positive result, some FSW who reportedly tested positive thought the HIVST result was inaccurate. This was especially so if previous HIV testing initiatives had produced negative results. *‘*…*Because at the hospital, they were finding me [HIV] negative’* (IDI, 11).

Conversely, an FSW who had previously undergone facility-based HIV testing and tested positive simply wanted to test reliability of the HIVST kit. *‘I just wanted to establish if it was different from the blood-based…I found that it was the same…If you have the virus, you have the virus’* (IDI, 18). Further probing revealed that doubts around HIVST, which in this study was oral-based, were largely due to failure to appreciate how HIV, perceived to be only found in blood, could be detected in saliva.

### HIVST, sex work and sexual partnerships

FSW described three types of male sexual partners: romantic boyfriends as well as temporary and regular ‘customers’, and recognised HIVST’s potential role in these partnerships. A relationship with a temporary customer was said to be largely transactional. FSW described a regular customer as someone they had known for a long time and the relationship transcended direct exchange of money for sex, although financial support was still an implicit requirement. *‘Because we started seeing each other and having sex a long time ago, we have reached a point of trusting each other. Whenever he is coming here, he phones to say, ‘book a room for me’, ‘cook for me’ because we love each other’* (IDI, 17). Trust and love were viewed as important ingredients of a regular relationship, even while financial support continued to be essential. Finally, respondents described a romantic boyfriend as one partner where the relationship is built on feelings, trust, love and not exchange.

‘Trust’ sometimes led to condomless sex within any longer-term relationships. This placed FSW at increased risk since they described how they sometimes felt disempowered to deny regular customers unprotected sex. ‘*There are some, you tell them ‘Aah here is a condom, put it on’ and they say, ‘I do not wear a condom’. That person is a long-time customer, right? It is like we end up referring to them as ‘‘my plain one’ [laughter]’* (IDI, 17). In this context, ‘plain’ sex means unprotected sex. Given this discourse of mutual ‘trust’, respondents felt that to the ‘plain clients’, they could possibly suggest HIVST as a precondition for having unprotected sex.

Of note, FSW expressed frustration over their inability to persuade both regular customers and romantic boyfriends to undergo facility-based HIV testing with them. *‘Some say, ‘I have a wife, so it is not good for me to go with you, better if it was my wife’…’* (IDI, 04). Respondents felt HIVST kits could potentially enable them to get these sexual partners to test. However, FSW also described occasional episodes of unprotected sex with temporary customers who offered to pay more (even when they were seemingly unwell). *‘There are some men who look unwell but want unprotected sex and say they will give you more money…’* (IDI, 14). Respondents wished they had self-test kits at the point of sex to enable them to immediately establish the HIV status of irregular partners requesting or demanding condomless sex.

### Linkage to post-test services

At the time of interviews, only three of the seven FSW that reportedly became aware of their HIV-positive status through HIVST had linked to treatment. In all cases, linkage to treatment was only reported in the repeat interview (held 3 months after HIVST). Reasons for the delay included reluctance, procrastination and lack of transport money. PDs expressed frustration over their inability to influence fellow FSW’s linkage to treatment *‘Some said they needed to think first before starting treatment. After 2, 3 days we went again, and she would say: ‘I am not ready to start treatment’. So, we ended up engaging some professionals [programme staff] as they kept giving us the same response’* (PD, FGD). Of note, those that reported treatment initiation said they accessed it from facilities outside their localities in order to avoid the stigma of being recognised. *‘I can say that the service I got is that I started receiving drugs. I access them in Zomba [55 km from Blantyre]. I don’t go to Blantyre because I am afraid that people might recognise me’* (IDI, 07).

## Discussion

This qualitative study suggested that barring a few shortcomings, offering HIVST to FSW through a peer-led model was largely feasible and acceptable. The study adds to the growing body of literature on the feasibility and acceptability of HIVST among FSW in SSA.^2 11 12^

Study findings are consistent with those from previous regional peer-based HIVST studies in general and those on populations often missed by conventional HIV testing services.^20–23^ For example, a recent study^23^ reported feasibility and acceptability of peer-based HIVST among men who have sex with men (MSM) in Uganda, a key population group that faces equally high levels of stigma and discrimination in the SSA setting. Feasibility and acceptability of peer-based HIVST have also been reported by studies exploring these issues among other mobile and HIV hyperendemic groups including fishermen.^20 22^ Collectively, these findings highlight the need to include these ‘hard-to-reach’ population groups in the planning, design and implementation of alternative strategies to conventional testing programmes.

The importance of peers in the context of feeling ‘other’ or different is well-recognised in programmes dealing with adolescents, FSW, MSM and among others^2 21^; there is great value in tapping into shared understanding and existing support strategies. Interventions targeting FSW and other key populations therefore need to optimise peer relationships and support networks, as multiple levels of influence not only exist but are also interactive and reinforcing.^24^ In fact, building and/or enhancing social cohesion among FSW is now recognised as one critical aspect of strengthening demand, improving supply, and supporting optimum use of HIV prevention or treatment strategies.^25^

Although our study has shown that encouragement to test is of particular value in promoting a peer-driven HIVST model, caution should be exercised to ensure peer-based HIV services (including HIVST) are non-coercive. Although peer pressure emerged rather anecdotally within this study, previous research has reported cases of FSW coercive testing.^2 26^ Importantly, programmes need to be aware of the vulnerability to peer pressure, especially of venue-based FSWs. This group is easier to access (which explains why about two-thirds of our IDI respondents were predominantly venue-based). This aspect exposes them to repeated research and programming, and ultimately, potential intervention fatigue. Further, power dynamics between venue owners and venue-based FSW require implementation of peer-based HIVST (or other HIV services) to take into account the multiple and multi-layered power relations that exist in the sex work environment.^16^ In addition to devising systems for monitoring social harms,^2 26^ HIVST programmes need to ensure the inclusion of structural and embedded mitigation approaches beyond simply ‘referring’ sex workers to external organisations.

Given the high levels of stigma and discrimination faced by FSW at public health facilities reported in this study and others,^27 28^ the requirement for FSW to continue accessing post-test services at these institutions remains a bottleneck within both the HIV prevention^29^ and treatment cascades.^30^ This likely explains why only a few HIV-positive respondents accessed treatment, undermining the benefits of HIVST. Alternative strategies could include empowering lay health workers acceptable to FSW to offer simple post-test services and establishing dedicated facilities. Of note, both dedicated services and HIVST may be particularly important for younger FSW who are less likely to visit public facilities or access other forms of testing.^31^ Dedicated services have demonstrated their effectiveness in reaching FSW of all ages in the SSA setting.^32–34^ Finally, continuing to address stigma at various levels (individual, community, facility) is critical for supporting reduction in HIV infections, regardless of the population group.^35^

FSW expressed doubts over the accuracy of oral fluid-based HIVST, with some describing retesting regardless of the result. While the failure to appreciate that HIV tests detect antibodies rather than infectious virus is understandable, programmes promoting HIVST need to explain this link more clearly and alleviate the doubts. This has the potential to enhance acceptability and uptake of this testing approach, which is now widely viewed as simple, less painful and less invasive.^2^ Programmes could offer both oral and blood-based HIVST as providing options can be beneficial.^36^ Use of HIVST among those already HIV-positive (and possibly on antiretroviral therapy (ART)) is a concern. Retesting while on ART has higher potential for negative results leading to misconceptions and confusion about HIV serostatus.^11^ As previously recommended,^11^ programmes need to engage with communities and targeted beneficiaries in developing clear HIVST messaging to reduce retesting while on ART in addition to addressing concerns and anxieties about the accuracy of HIV-positive diagnoses.

Worryingly, FSW described instances where they had condomless sex within all types of partnerships, often without knowledge of their partners’ HIV serostatus. HIVST may be viewed as a testing modality with the potential to lead to FSW’s greater sexual negotiation power and reduced risk behaviours.^37^ For example, it can facilitate other harm reduction strategies including point-of-sex HIV testing^38^ and serosorting (where individuals limit their sexual partners to those with the same HIV status as themselves).^39^

This first HIVST study among FSW in Malawi was integrated within an established FSW service. The study reinforces the value of HIVST in key populations subject to stigma when accessing standard services. There was concordance between our study findings and those from other studies, including around FSW’s low rates of linkage to treatment, which suggests that the context in Malawi is not unique. However, we used convenience and snowball sampling to select respondents, which, while more feasible in populations with potentially stigmatised behaviours, may not be universally representative. Additionally, given the fluidity of sex work typologies of street or venue-based FSW in this population, it was not possible to attribute study findings to specific typologies. The use of male interviewers potentially created gendered power disparities between researchers and FSW. The richness of our transcripts, however, illustrate that FSW freely discussed with the male researchers who were both experienced and trained to effectively manage power relations with study participants. Further, we had intended to re-interview all IDI respondents after 3 months but only managed to reach 28% due to FSW’s recognised mobility.^22^ Lastly, we could have ensured better involvement of PDs in data analysis in line with enhancing stakeholder involvement. Despite these limitations, our results are important for informing adaptation of HIVST delivery approaches to the needs of key populations in general and FSW, specifically.

## Conclusions

This study enabled us to explore the feasibility and acceptability of peer-led HIVST within the context of FSW in three settings in Malawi, and highlight the potential limitations of this HIVST approach. We found that HIVST has several benefits for FSW in general and younger FSW, specifically. Peer distributors are a welcome additional model. However, they should avoid distribution in actual venues due to the pre-existing power imbalances between the venue owners and FSW. Additionally, programmes should ensure a range of testing options are available (eg, community based) to avoid coercion and retesting out of desire to please the PD. Further, while peer-led approaches are valuable in targeting FSW involved within tight-knit social networks, inclusion of peers representing those not attached to venues prior to implementation will help to reach those FSW who may be more vulnerable. Finally, unless alternative linkage approaches are put in place, the requirement for FSW and other key populations to seek post-test services at public health facilities remains a bottleneck within both the HIV prevention and treatment cascades.

## Data Availability

Data are available on reasonable request. Anonymised participant data can be made available on reasonable request to the corresponding author at webster@ceshhar.co.zw.
