# Supplementary material for: Feasibility and acceptability of a peer-led HIV self-testing model among female sex workers in Malawi: a qualitative study
Source: BMJ Open. 2021 Dec 30;11(12):e049248. doi: 10.1136/bmjopen-2021-049248 (PMC8718425; doi:10.1136/bmjopen-2021-049248)
Supplement: Supplementary data [file bmjopen-2021-049248supp001.pdf]

PS903: INTERVIEW GUIDE

Malawi-Liverpool-Wellcome Clinical Research Programme
P O Box 30096, Chichiri, Blantyre 3, Malawi
Tel: +265 1876444 ▪ Fax: +265 1875774 ▪ www.mlw.medcol.mw

Serial Biographical Interview Guide

Section 1: Participant-led biography

1. Ask the participant to describe a positive memorable experience in their relations with others that happened in the previous 3 months in their household in which they were involved
2. Why was this memorable? (Probe: frequency, representativeness of event)
3. Ask the participant to describe a negative memorable experience in their relations with others that happened in the previous 3 months in their household in which they were involved
4. Why was this memorable (Probe: frequency, representativeness of event)
5. Ask the participant to describe their family and home life in general over the last year (Allow participant to focus on what they think is important but probe: family dynamics i.e., comings and goings, relationship, family relationships with examples)

Section 2: Home environment

6. Can you describe your current relationship? (Probe: length of relationship, number of children, marital status)
7. Can you describe for me the positive and negative aspects of your relationship with your current partner? (Probe: what is good about your relationship? What is bad about your relationship? Move beyond economics!)
8. Can you describe your emotional relationship with your current partner? (Probe for details on multiple partners, infidelities on both sides that may have precipitated event)
9. Can you describe your physical relationship with your current partner? (Probe for details on multiple partners, infidelities on both sides that may have precipitated event, previous incidents of violence)
10. What kind of things are hard to discuss with your current partner?
11. What kind of things are easy to discuss with your current partner?
12. How long do you plan to stay with your current partner? Why?
13. Have you ever experienced violence or been violent in previous relationships? What impact did this have on your previous relationship? What impact do you think your previous experience of violence has had on your current relationship?

Section 3: General household decision-making

14. Can you describe what responsibilities you have in your home? Do you think these responsibilities should all be yours? (Probe: decision-making and circumstances)
15. Can you describe what responsibilities your partner has in your home? Do you think these responsibilities should all be his/hers? (Probe: decision-making and circumstances)

Section 4: Decision-making around HIV testing and self-testing

Introduce the next questions by stating: Persuading someone to test involves discussion and the person makes the final decision to test. Forcing someone to test involves emotional or physical abuse and often the person does not make the ultimate decision to test.

16. Have you ever been persuaded to test for HIV? (Probe: in which relationship, previous or current, how did they persuade you, why did they persuade you, how did this relate to your general relationship with this person? Was it typical behaviour?)
17. Have you ever been forced to test for HIV? (Probe: in which relationship, previous or current, how did they persuade you, why did they persuade you, how did this relate to your general relationship with this person? Was it typical behaviour?)
18. Did you speak to anyone else about your decision to test for HIV (before self-testing)? (Probe: who did they speak to (relationship), why, how did this help?)
19. How positive or negative was your previous experience of testing? (Probe: what were the positive and negative aspects with regard to the results, their relationship with partner and others)
20. Was this previous testing experience with your current partner or a previous partner? What was their reaction?

Malawi-Liverpool-Wellcome Clinical Research Programme
P O Box 30096, Chichiri, Blantyre 3, Malawi
Tel: +265 1876444 ▪ Fax: +265 1875774 ▪ www.mlw.medcol.mw

21. Have you ever tested with anyone else? (Probe: couples testing, testing with friends, if yes, how this improved the experience)

Can you describe your recent experience of self-testing with STAR? (Probe: What were the circumstances of testing? Ask the participant to describe the experience in full in their own words before moving on to more specific questions)

22. Were you with the same partner who you previously tested with when you self-tested this time? What was their reaction to this self-testing?
23. Who initiated testing including partners/STAR counsellors/neighbours etc.? (Probe: How far do they feel they were influenced to test? How far do they feel they were persuaded to test? How far do they feel they were forced to test?)
24. Who collected the test kit(s) or were they delivered? How many test kits did s/he bring home/receive?
25. What do you feel was the main reason why you self-tested with HIV?
26. How much did you feel personally that you 'needed' an HIV test? (Probe: perceptions of risk and assessment of previous sexual risk behaviour)

Section 6: Responses post self-testing including risk behaviour

27. After testing and reading the results what happened? (Probe: disclosure (to whom and under what circumstances), force/persuasion of others to test including family members and partners)
28. Was your result as expected? Why? Why not?
29. Is your HIV status the same as that of your partner? (Probe: Did you expect it to be the same? Why? Why not?)
30. If you found out your partner's results, were they as expected? Why? Why not? (Probe: What was your reaction?)
31. If your partner found out your results, were they as your partner expected? Why? Why not? (Probe: What was their reaction?)
32. How has your relationship with this partner changed since self-testing? (Probe: positive and negative aspects of change, get them to think about physical, relational, emotional, changes including attitude towards the participant)
33. How has your sexual relationship with your partner changed since self-testing? (Probe: trust, condoms, frequency, discussions around sex, other partners, perceptions of risk?)
34. Do you feel better or worse about your relationship after self-testing? Why? Why not? (Probe: power relations within the household and position of participant within these)
35. How has your relationship with other people in your home changed after self-testing? (Probe: responses from different family members, power relations within the household & impact on disclosure and post-disclosure experiences)
36. How much do you think the changes you have described are due to your being a woman/man (Probe: explore how they think their gender impacted on relations after self-testing)
37. Have you felt in danger of being verbally or physically or sexually abused with either your current or any other partners since self-testing? Probe: Why or why do they not think they have been at risk of violence)
38. Have you been hit/kicked or hurt by your partner or anyone else since self-testing? (Important probe: whether they think this is due to the self-testing)
39. Do you think it is acceptable to force someone to take an HIV test? In what circumstances?
40. Do you think violence is acceptable under certain circumstances? In what circumstances?

Kumwenda MK, *et al. BMJ Open* 2021; 11:e049248. doi: 10.1136/bmjopen-2021-049248
